# A double narrative: stories underpin fearful community attitudes toward snakes but offer a mechanism to reduce human-to-snake conflict

**DOI:** 10.1080/16549716.2026.2655948

**Published:** 2026-05-05

**Authors:** Diana Meirinho Domingues, Kevin Arbuckle, Hazel J. Nichols, Yowasi Byaruhanga, Jessica Mitchell

**Affiliations:** a Edinburgh Medical School, College of Medicine and Veterinary Medicine, University of Edinburgh, Scotland, UK; b Department of Biosciences, Faculty of Science and Engineering, Swansea University, Swansea, UK; c New Life Junior School, Bushenyi, Uganda; d Global Agriculture and Food Systems, College of Medicine and Veterinary Medicine, Easter Bush Campus, Midlothian, University of Edinburgh, Scotland, UK

**Keywords:** Storytelling, snakebite, community engagement, behaviour, Uganda, qualitative methods

## Abstract

**Background:**

Snakebite envenoming is a neglected tropical disease with significant mortality and morbidity. Fear of snakes often results in human-to-snake violence which not only harms snakes but increases bite risks to humans. Limited research exists on both the origin of these negative attitudes, and strategies to promote human-snake coexistence.

**Objectives:**

Using secondary qualitative analyses, we explored the role of snakes within community stories, in Southwest Uganda. We investigated the influence of stories on community attitudes towards snakes, and how these attitudes have changed since engaging in participatory snakebite prevention workshops which included narrative discussions.

**Methods:**

In 2022, snakebite prevention workshops were delivered, followed by semi-structured interviews (*n* = 21) of workshop attendees and non-attendees. These interviews included questions about the content of local snakebite stories. In this study, a secondary analysis using inductive reflexive thematic methods was performed to specifically consider the relationship between local stories and community attitudes toward snakes.

**Results:**

Attitudes toward snakes were largely fearful. Hearsay and stories appear to influence this fear and general knowledge of snakes in this community. Stories usually depicted snakes as villainous, supernatural, or malevolent. However, participatory snake-bite prevention workshops which include narrative discussion and two-way knowledge exchange with participants appeared to support attendees to change their attitudes and behaviours toward snakes.

**Conclusions:**

Stories appear to influence community attitudes toward snakes in Rubirizi. Future research should focus on understanding local narratives around snakes, and harness the power of storytelling to softly challenge these narratives in order promote human-snake coexistence and snakebite prevention.

## Paper context

- **Main findings:** In rural Uganda, local stories influence negative attitudes toward snakes which can in turn drive human-to-snake conflict, a major cause of snake bite at community level.
- **Added knowledge:** Participatory workshops on snake bite prevention appear to provide a feasible route to challenge negative community attitudes toward snakes and promote human-snake coexistence.
- **Global health impact for policy and action:** Future policy and practice should encourage the incorporation of local knowledge into snake bite prevention interventions, specifically, story-telling and narrative approaches could be harnessed to effectively challenge snake-related fear, reduce human-to-snake conflict and ultimately prevent snake bites.

## Background

Snakebite envenoming is a neglected tropical disease contributing to around 140 000 annual global deaths plus morbidity arising from lifelong injury and post-traumatic stress [1]. Snakebite is unequivocally a disease of poverty [2], with the highest individual-level risk factors including engagement in agricultural activities. At population level, the incidence and sequelae of snakebite are impacted not only by proximity to venomous snakes but also access to medical treatment and travel time to reach such treatment. A comprehensive analysis of such factors revealed that most snakebite vulnerability hotspots were in Central and Eastern Africa [3], with South-East Asia a close second. Meanwhile, the burden associated with snakebites, especially in Africa, is under-appreciated relative to other neglected tropical diseases [4,5].

The ecology of snakebite research is similarly complex with many studies focused on refining medical snakebite treatment or training and access to such treatment, as well as encouraging the uptake of hospital treatment at community level [3–10]. However, the focus on treatment is unsustainable, especially within low resource settings such as central and eastern African areas which are disproportionately vulnerable to snakebite [3]. In such contexts, treatment centres are often poorly resourced, difficult to access, and confined to urban areas [6]. Moreover, successful treatment may still leave victims with debilitating mental and physical sequelae such that, especially in vulnerable areas, snakebite prevention appears a much more sustainable intervention strategy [7,8]. Bite prevention not only benefits the human population but can also have conservation and ecological benefits by protecting snakes from human violence, which is often an immediate response following a (suspected) bite or even simply encountering a snake [9–11].

Snakebite prevention is gaining attention at global policy level, with the World Health Organization (WHO) releasing a new strategy to halve snakebite envenoming by 2030 [12]. A key pillar within this strategy is ‘Engaging and Empowering’ communities to prevent snakebite. The document calls for a biosocial approach to understand the context in which snakebite occurs [9]. Such calls are mirrored within the recent, if limited, academic literature on snakebite prevention, with some research focusing on the need to actively engage communities with strategies using local languages and accessible forms of communication [13].

Our authorship team have been working in the Rubirizi community of South-Western Uganda since 2022 to explore knowledge, attitudes, and practices around snakes, snakebite prevention strategies, and treatment. Our work has centred on the delivery of snakebite prevention workshops which seek to exchange knowledge with communities and generate meaningful action points to prevent snakebites [14]. Many anecdotes exchanged in these workshops are feature local stories or hearsay around snakes. The value of oral stories is high across Uganda and is commonly seen to process the past and problem solve, often via making use of the wisdom of elders [15]. Such findings are common across multidisciplinary research into the value of stories or narratives [16–18]. Storytelling is seen as a way to process one’s identity, problem solve, imagine and emotionally adapt to future scenarios, but also generates attitudes and opinions on the story topic. As such, we hypothesise that stories may have a significant impact on community attitudes toward snakes and that understanding this impact could allow refinement of future workshops and snakebite prevention strategies.

This study is a secondary analysis of existing qualitative data, through which we asked:

1. What roles do snakes take in local stories from the Rubirizi area?
2. What is the influence of these stories on community attitudes toward snakes?
3. How do Rubirizi community members consider their attitudes and behaviours toward snakes have changed since engaging with snakebite prevention workshops?

By exploring these research questions, we aim to suggest mechanisms to refine future snakebite prevention interventions with a focus on changing negative attitudes toward snakes.

## Methods

### Study site and context

This study was conducted in the Rubirizi District of Southwest Uganda, a region including part of the Ugandan Wildlife Authority governed Queen Elizabeth National Park. Population size is estimated around 144,100 people [19]. Settlements are predominantly rural, with most households owning land and subsistence farming accounts for 69% of the region’s income [20]. Many households also gain seasonal income from tourism and National Park employment. Due to high levels of outdoor employment, snake encounters are common, yet there is currently very limited information on snake species present in this area. Instead, knowledge of snakes and snakebite risk is almost exclusively held at community level. Engagement with such lived experience data is likely to be essential in shaping snake bite prevention strategies and this has been the impetus for our authorship teams’ work. Treatment for snakebites is variable across Uganda but generally considered to be weak, with minimal medical training on snakebite envenoming and poor access to effective snakebite treatments [21–23]

### Previous engagement with this community

The authorship team has worked with the Rubirizi community since 2022 to explore snakebite prevention approaches. From May–October 2022 a series of snakebite prevention workshops were designed and tested in this community. Workshops took a narrative and participatory approach involving community members in classroom and outdoor activities to share knowledge regarding snakes, snakebite prevention, and treatment. To understand the impact of workshops, a survey and 21 in-depth interviews were conducted in May 2023. The workshops are referenced throughout the interview questions, resulting transcripts, and this manuscript. For a full overview of the workshop format, see the supplementary file, and for local knowledge, attitude and practice data regarding snakes, see Kevin Arbuckle and Kaseke [14].

### Study design

This study employed secondary thematic analysis to specifically explore the role of snakes within local Rubirizi stories, as well as overall attitudes toward snakes. This approach was taken as the value of story-telling was apparent but could not be captured in detail, within the authorship team’s original Knowledge, Attitudes and Practice (KAP) analysis [14]. Secondary analysis methods do face criticism, mainly due to a lack of background information and transparency regarding the aims of the original study [24]. In this manuscript, authors have described the original study in as much detail as possible, including all participant engagement methods, researcher roles and ethical considerations.

### Original recruitment

Interview recruitment was via word-of-mouth spread by YB; a member of the research team, local primary school director and respected community leader. YB shared the interview aims and topics, participants’ right to withdraw, and details surrounding data protection. Much of the community were already sensitised to the project having been recently involved in snakebite prevention workshops. However, recruitment for interview targeted the whole community, including those who had not previously attended workshops. YB ensured all members of the community including men, women, professionals, homemakers, farmers, faith leaders, community gatekeepers and village health teams were aware of the interviews. Interested persons could book a timeslot to meet YB and KA for the interview. Twenty-one participants were interviewed, giving a relatively large sample size for a qualitative study and likely to reach saturation [25]. Ages ranged from 18 to 50 though many did not know their exact age. Educational backgrounds varied from individuals who had not completed primary school to diploma graduates. Anonymised participant data is displayed below (Table 1), with further information available within the supplementary file.

**Table 1.** Summary of anonymised participant data.

Transcript Number	Gender	Relation to snakebite workshop
1	Female	Attended workshop
2	Female	Did not attend workshop, though heard of it from word-of-mouth
3	Female	Did not attend or hear of workshop
4	Female	Attended workshop
5	Male	Did not attend or hear of workshop
6	Male	Attended workshop
7	Female	Did not attend or hear of workshop
8	Female	Attended workshop
9	Female	Attended workshop
10	Female	Attended workshop
11	Female	Attended workshop
12	Female	Attended workshop
13	Male	Did not attend workshop, though heard of it from word-of-mouth
14	Female	Attended workshop
15	Male	Did not attend workshop, though heard of it from word-of-mouth
16	Male	Did not attend workshop, though heard of it from word-of-mouth
17	Female	Did not attend or hear of workshop
18	Female	Did not attend workshop, though heard of it from word-of-mouth
19	Female	Attended workshop
20	Female	Did not attend workshop, though heard of it from word-of-mouth
21	Male	Did not attend workshop, though heard of it from word-of-mouth

### Original data collection

Semi-structured interviews were conducted with 14 pre-set questions (see supplementary file). The interview guide was developed by JM, KA, HN, and YB and explores areas including participants’ knowledge, attitudes and experiences relating to snakes and snakebites, including local stories surrounding snakes, plus information retained from recent snakebite prevention workshops, where applicable. Interviews were conducted by YB and KA in a public space easily accessible to participants. Interview questions were delivered in English by KA and then translated by YB into local languages if required, with YB fluent in English, Luganda, and local tribal languages. At the point of interview, the aims of the project and rights to withdraw were reiterated. All interviewees provided verbal consent, captured in the interview recording. Interviews were recorded on a Dictaphone (Evistr L157 16Gb Digital Voice Recorder), then transcribed and translated verbatim into English by YB without additional software. Final transcripts were anonymised and recording files stored on JM and KA’s secure university accounts and deleted from the original Dictaphone.

### Reflexivity

To ensure participants felt comfortable sharing their experiences, YB conducted all interviews (with KA) and transcribed and translated the data. However, we recognise that YB holds a position of power as a school director and community leader which could influence participants’ answers or willingness to participate. YB kept notes regarding the ‘feel’ of interviews and noted any areas in which people were reluctant to speak, corrected themselves, or changed their stories. Such moments were rare and mainly attributed to nerves which were mitigated by telling participants that honest answers are helpful and that the interview was a safe space. KA (Scottish researcher and herpetologist) was also present and active within interviews. This was mainly to ensure any snake biology, safety, or behaviour questions could be addressed by a specialist to prevent harm due to misinformation. However, the team balanced this against the risk of KA’s presence reducing participant engagement. Based on KA’s and YB’s interview reflections, KA’s presence did not appear to impact engagement and in many cases, participants were comfortable to speak in English and addressed workshop feedback directly to him. KA does not understand local languages and we believe this may have contributed to a feeling of safety for participants to openly answer questions in languages other than English where necessary.

### Data analysis

Qualitative interview data were analysed using Braun and Clarke’s approach to inductive reflexive thematic analysis [26]. This permits the identification and reporting of key themes and sub-themes within the data and reflection on their pertinence to the research questions. Two researchers (DMD and JM) read the interview transcripts repeatedly resulting in the identification of seven main themes and 36 potential sub-themes. The two conferred extensively, collapsing themes with areas of overlap and agreed upon a final set of six key themes and 21 sub-themes. Using themes as emergent codes, they built a coding frame (Table 2) to guide the analysis process. All coding was completed in NVivo 14.0 software. Coding was initially performed by DMD and reviewed by JM.

**Table 2.** Coding frame used in the inductive thematic analysis of the 21 interview transcripts.

Theme	Sub-theme	Link to research questions
Attitudes toward snakes	Understanding	1, 2
	Uncertainty	1, 2
	Respect and coexistence	1, 2
	Importance	1, 2
	Hate	1, 2
	Fear	1, 2
	Danger	1, 2
	Curiosity	1, 2
Behaviours toward snakes	Ways of treating snakebites	2, 3
	Ways of avoiding snakebites	2, 3
Double code	Before attending the workshop	3
	After speaking to a person who attended the workshop	3
	After attending the workshop	3
Sharing of knowledge	Sharing information from workshop	3
	Hearsay from community	2
Symbolic perceptions of snakes	Villain	1
	Threat	1
	Supernatural	1
	Saviour	1
Violence	Violence of snakes toward humans	2
	Violence of humans toward snakes	2

## Results

### What roles do snakes take in local stories from the Rubirizi area?

Eighteen stories were shared by participants (Table 3). Aside from the biblical story, not a single story was repeated, but several key themes appeared consistently across stories.

**Table 3.** A summary of the specific snake stories described by Rubirizi community members.

Participant	Story/folklore summary	Key themes
1	Three individuals sought shelter from the rain inside a hut. The person inside offered them meat, which one of them accepted. The person then turned into a snake and ate the individual that consumed the meat.	Supernatural Villain Snake-to-human violence
2	Folk tale describes snakes chasing people, hence preventing them from accessing forests and bushy areas.	Villain Threat
4	Children went alone to fetch water from a lake. There, they found a snake. The children returned home without water as they were scared.	Threat Fear
5	An individual went to a witch doctor, wanting the hand of a girl in marriage. The witch doctor sent a snake, who threatened to eat the girl unless she accepted the man’s marriage proposal.	Supernatural Villain Threat
5	A person was concerned that their business partner was corrupt. They went to a witch doctor, who sent a snake to burn the corrupt person’s house down.	Supernatural Villain Threat Snake-to-human violence
5	An individual had their bike stolen and went to a witch doctor. As the witch doctor practiced their craft, a snake passed by. According to the participant, the snake is indicative of magic and witchcraft taking place.	Supernatural
6	A snake sought shade from the sun, smelled food and was attracted to someone’s house. There, it was discovered by humans, killed, and buried.	Human-to-snake violence
7	The Rat Eater (Ekirya Mbeba) is described as a ‘house protector’. This snake is claimed to be non-venomous and docile, protecting the house from rodents that would otherwise eat the household’s food.	Saviour
7	When people still used pots to fetch water, the black spitting cobra is described as jumping out from the trees and landing in the pots to drink said water, leading the pots to burst.	Threat
8	A girl is described as being lazy, not wanting to carry firewood and reticent to cross a river. Hence, this girl accepts a snake’s help. The snake then follows her to her village and hunts her down.	Supernatural Threat
10	An old woman sees a snake in her house. She initially hates the snake, but then sees it eat and eradicate the rats from her house and recognizes the snake’s importance. They proceed to live in harmony.	Saviour
10	In the Bible, the snake provokes Adam and Eve in the Garden of Eden. Since then, this paints a residual ‘bad image’ about snakes.	Supernatural Villain
14	As described in the Bible, the snake provokes Adam and Eve to eat the forbidden fruit.	Supernatural Villain
15	In this family, they believe that when someone thieves from the property, a snake will come and capture the thief and bring back the stolen entity.	Supernatural Saviour Threat
16	When this person went fishing near a lake, they found a large snake near their house and felt fear. They later learned from the community that this was a demonic snake, referred to as the ‘king of the lake’.	Supernatural Fear
19	A snake wanted a girl’s hand in marriage, and she refused. When the girl inadvertently later sought shelter from the rain in the snake’s cave, the snake blocked the exits until the girl accepted the snake’s marriage proposal. The snake then turned into a very handsome man, and they got married.	Supernatural Threat
19	Some people cut their skin and apply shed snakeskin onto it, as this grants longevity.	Supernatural
21	People were trying to kill a satanic snake. The snake jumped into the water, leading everyone chasing it to fall in as well.	Supernatural Villain Violence

#### Fear (general)

Fear of snakes is salient throughout the interviews, with 11 participants explicitly stating that they feared snakes, 11 participants verbalising hate towards them, and 19 describing them as dangerous.

#### Fear (of the unknown)

Knowledge about snakes, especially before the workshop, seems heavily reliant on hearsay from community members. Interviewees describe how snakes chase people (Participants 2, 5, 13, 15), spit out stones (Participant 13), grow horns (Participant 6), and that snakes’ tails can bite and/or are venomous (Participant 6, 8, 16). Some participants (6, 13, 16) are not describing their own fears but recalling fears of the wider community. Their answers begin with ‘some people say … / they told me”, with some participants suggesting they do not always believe these stories.

Some people say that some snakes have venom on their tails, so I don’t believe that is right, there are some who say some snakes grow up they develop horns I think that one is also false (Participant 6)

There is also uncertainty about whether snakes can be eaten (Participants 1, 12), whether they produce mushrooms (Participants 5, 21), and whether humans can hold them without being bitten (Participant 20). This uncertainty around snake behaviour and ecology appears to accentuate the fear and distrust toward snakes (Participants 17 and 20).

I hate them, and I don’t like them because I haven’t learnt about them (Participant 17)

#### The supernatural

Within Rubirizi stories, snakes are often described as supernatural. Some turn into humans and vice-versa (Participants 1, 19), witch doctors use snakes to deliver threats (Participant 5), and snakes are often depicted as threatening characters (Participants 1, 8, 15).

When the rain stopped the girl turned into a snake, into a very big snake which went into the doorway and blocked it (Participant 1)

In most tales, the snake is often in a position of power; occasionally referred to as ‘satanic’ (Participant 12) or ‘demonic’ (Participant 16). Perceiving snakes as supernatural also extends beyond stories. Participants 2 and 3 described people having snakes in their house correlating to those people owning ‘some demons or small gods’ (Participant 2). When asked for clarification, participants ascertained that this was viewed negatively (Participant 3). However, other participants (Participants 1, 4, 10, 16, 18, 19, 21) referenced snakes being used as *totems* which occurs when supernatural good fortune is attributed to a particular animal. Where a family has a snake as a totem, they believe it may offer them protection or luck and, in such cases, snakes were described more positively.

[totems are] traditionally when something has either saved or done something strange the family decides to put it as a totem either in a good way or bad way but people align to it and do not harm it as a totem (Participant 19)

#### The villain

Snakes are frequently portrayed as villainous characters, having a fearsome, threatening presence. For example, snakes are often depicted as blocking doorways (Participants 1, 19), chasing (Participant 2, 8, 15), attacking (Participants 1, 5) or threatening to eat (Participants 1, 5) people.

There are still stories that sometime back they would meet snakes, they would chase them, that would limit us from visiting forest and bushy areas (Participant 2)

There are also several references of snakes forcing girls into marriage (Participants 5, 19).

‘The witchdoctor said to them, if they pay him, he will send a snake to the girl. So, the snake will say ‘If you don’t accept me then I will eat you’ […] And then the lady, the girl, accepted to get married to the tiny boy because the alternative was to get eaten (Participant 5)

#### The saviour

In some cases, snakes take a more positive role in the stories, acting as saviours. However, the snake’s protective function often stems from its aggression and reinforces the general theme of fear (Participant 15). Alternatively, the snake’s ‘saviour act’ (e.g. offering food or aid) later results in the snake hunting down the protagonist as retribution (Participants 1, 8).

We have a belief that a snake will go capture somebody who comes and steals from our property (Participant 15)

A few participants mention the snake in a wholly positive light within their folktales, mainly protecting the house from rodents (Participants 7, 10).

When the old woman would find snakes in the house eating rats, she would feel happy because it was eradicating rats from the house (Participant 10)

This extends to most people having some knowledge of the positive attributes of snakes to the community. When asked directly, participants named benefits including rodent control (Participants 1, 3, 6, 7, 9, 11, 14), food for animal (Participant 5) or human consumption (Participants 10, 14), research (Participants 6, 8, 13, 14, 19, 20), tourism (Participants 6, 7, 8, 9, 13, 14), medicine (Participants 8, 10, 13, 15, 19), and making clothes or instruments (Participants 1, 3, 4, 6, 9, 10, 11, 12, 13, 14, 21).

[Snakes] are important and have good qualities. They bring in tourists, from their skins we get shoes, and making drums. They eat rats from the house and gardens (Participant 9)

### What is the influence of these stories on community attitudes toward snakes?

#### Perceived danger (fear)

Fear of snakes often appears linked to their perceived danger (Participants 2, 3, 18).

I don’t love them in any way because they are venomous and all snakes are venomous and when they see me, they might bite me so I hate them (Participant 18)

There were minimal reports of deaths due to snakebites (one death was reported by Participant 2), with the majority of snakebite victims being ‘okay’ or ‘in good health’ irrespective of the treatment provided (Participants 1, 5, 6, 7, 9, 10, 11, 14, 16, 17, 18, 19, 20, 21).

Additionally, participants collectively described seeing snakes in non-threatening positions, ‘relaxing’ (Participants 1, 5), ‘coiling’ (Participants 1, 2, 3, 5, 7, 9, 10, 11, 14, 16, 18, 19), or ‘running away’ (Participant 3, 4, 5, 6, 7, 13, 15, 17).

Some are always coiling and relaxing under the something, some others are moving. […] They’re always running away (Participant 5)

Nevertheless, two individuals report interpreting these seemingly docile positions as threatening, thus perceiving snakes more aggressive than they otherwise may be.

When you find it coiled it means it has too much venom and very dangerous (Participant 8)

#### Warning

Snakes roles within stories can lead to community members associating snakes with warnings (Participant 8). However, some stories leverage this existing fear of snakes to warn about other dangers. For example, Participant 4 describes a story where children went to get water from the lake and were chased away by snakes.

The stories are not true; they are just to scare the kids from going alone to fetch water without an elder person (Participant 4)

#### Malevolent

Most participants describe snakes as ‘wanting’ or ‘needing’ to bite humans. We coded this as ‘Malevolent’ to capture the community’s anthropomorphism suggesting snakes have a specific will to harm.

When it sees you before you see it, it’s always targeting to bite you (Participant 15)

This perceived malevolence, in turn, appears to drive community aggression toward snakes.

I just kill them when I see them because when they see me, they will only bite me (Participant 16)

### How do Rubirizi community members consider their attitudes and behaviours toward snakes have changed since engaging with snakebite prevention workshops?

#### Change (in fearful attitudes)

Workshop attendees continue to fear snakes but discuss how learnings from the workshop have supported them to change their aggressive behaviours toward snakes.

Though am still scared of them but I just give them peace and they go away (Participant 6)

#### Change (in practices and behaviour)

Many described learning new ways of avoiding snakes near their home, have begun using resources that help prevent snakebites (e.g. mosquito nets, boots, and lights), and have altered some incorrect assumptions they may have had about avoiding snakes prior to the workshops. For example, Participant 17 discusses that before the workshops:

To prevent the snakes for us who live near the forest our mum told us that we always use buckets in the house and we don’t cover them because when a snake comes and it’s thirsty it will drink the urine and it will lose the morale of biting somebody (Participant 17)

Meanwhile, after the workshop participants learnt to:

Always cover the buckets containing their urine at night because the snake might be thirsty needing water and then enter the house and bite them (Participant 8)

Several participants described doing some preventative actions prior to the workshops, and these behaviours were reinforced.

Everything changed after the workshop. I used to clean but not knowing I was preventing snakes but now I do it even more because I did not know that the holes are snake habitats (Participant 19)

Information and change of attitudes also spread to people who did not attend the workshop through word-of-mouth.

We used to clean our house and the rest for purposes of hygiene but when our dad [who attended the workshop] came it was improved more with same methods even preventing snakes (Participant 13)

#### Change (in human-to-snake violence)

Workshop attendees describe a decrease in their own human-to-snake violence. Many explicitly said that after attending the workshop they modified behaviours such as upon seeing a snake they would give it space and let it go on its way (Participants 1, 4, 6, 8, 9, 10, 11, 12, 14, 19) or give it peace (Participants 8, 10, 12, 14), whereas before, they used to attack or kill it (Participants 1, 4, 6, 8, 9, 10, 14, 19).

Before the workshop I used to hate snakes and then kill them every time …

From the knowledge of the workshop I give [the snake] its peace, if it’s in the road I just cross via the shortcut and then go away without tampering with it (Participant 8)

However, non-attendees who had heard about workshops from word-of-mouth state that they would still attempt to kill snakes (Participants 13, 16, 18).

The option would be killing it if it was small and if its big, I will just run away (Participant 13)

## Discussion

### What roles do snakes take in local stories from the Rubirizi area?

When participants were asked about stories featuring snakes, most could recall at least one. These stories often depicted snakes as evil, with sinister intentions, associated with the supernatural. This is similar to popular folklore across Uganda, wherein snakes feature prominently in witchcraft tales, harbouring aggressive and malevolent attributes [27]. This is common throughout cultural history, including texts from Hinduism, Judaism, Greek, and Egyptian mythology where snakes hold harmful, fear-inducing roles [28]. The villainization of snakes is noted across other communities, including those in Ghana, Nigeria, Bangladesh, and Brazil [10,29–31]. In these examples, snakes often hold supernatural or anthropomorphic qualities including the ability to speak, morph into human forms, and wider abilities beyond natural snake behaviours.

Most stories recounted by the Rubirizi community held positive morals similar to how folktales are used worldwide to guide, inform, and protect communities [32,33]. Occasionally, Rubirizi stories position snakes in a saviour role, often personifying it as a vengeful guardian. This is similar to how feared species are viewed throughout other cultures, including the threatening yet protective depiction of wolves and tigers in Roman and Chinese culture, respectively, [34,35]. In our data, some stories describe the snake as a ‘house protector’, useful in managing the rodent population, which is more akin to snakes’ natural, observed behaviours. These beneficial attributes are predominantly discussed by workshop participants, or those who have spoken to workshop participants. They mirror some of the points made by community members and reinforced by the research team within workshops. Workshop facilitators created a pro- and con- list of snakes, and protective behaviours around home hygiene and rodent killing were frequently suggested by community members and discussed at length in the workshops. Facilitators used this community knowledge as a ‘hook’ to advocate for snakes and avoidance of human-to-snake violence [16,17]. It is encouraging to see that post-workshop the benefits of snakes are still being discussed at community level and stories supporting this narrative are recalled. By building upon recognised narratives (i.e. snakes are beneficial), future snakebite prevention interventions can more effectively explore avenues for behaviour change which are already palatable to the community.

### What is the influence of these stories on community attitudes toward snakes?

Community attitudes surrounding snakes are largely fearful, a sentiment ubiquitous in the literature [10,36,37]. In Rubirizi, this appears to prompt human-to-snake violence. Participants who had not attended a snake bite prevention workshop considered snakes inherently aggressive towards humans, with malicious intent to chase, bite, and kill. Therefore, killing snakes is described as imperative to avoid later being hunted oneself. Viewing snakes as naturally malevolent is similar to how snakes were portrayed within local stories. This belief, held strongly in Rubirizi, is not always echoed across other populations, with some similarly rural Kenyan communities viewing snakebites as simply unlucky and unpredictable [37]. This emphasises the importance of understanding local context, including stories, if aiming to design interventions to reduce human-to-snake conflict.

Despite fearful attitudes in the community, personal recollections of fatal snakebites are limited, with only one participant reporting such an incident. This is supported by a mixed methods survey conducted within Rubirizi in 2022–23 [14]. Here the community reported six fatalities amongst their recollections of 178 presumed snakebite incidents, over the past 2 years. It is important to note that these data are based on personal recollections and not medical reports confirming snakebites, nevertheless they serve to highlight that Rubirizi community members have limited personal experience of snakebite mortality despite their explicit danger-based fear of snakes. This is not surprising based on the global snake perception literature, wherein many studies have explored whether the snake fear response is innate or a learned reaction [38,39]. Most suggest that while there may be evolutionarily preserved neuronal pathways leading to fast reaction times in recognising snakes compared to non-threatening stimuli, this does not correlate with increased levels of fear [40]. Rather, associating snakes with fear is most likely learned via stories from community members and the media [36,41,42]. Our analysis reinforces that stories are important contributors to human-to-snake conflict. However, snakebite can have severe, non-lethal impacts on human health and wellbeing including limb loss, post-traumatic stress disorder, loss of earnings etc. Thus, recollections of 178 suspected snakebites in the past 2 years likely represent significant trauma for this community, even though most victims survived.

In Rubirizi, stories seem to play an influential role in attitudes towards snakes. This is unsurprising considering wider research exemplifying that in Uganda stories are essential communicative avenues for problem-solving in real-time and imagined futures [43]. Unfortunately, the prevailing negative roles of snakes within stories make it likely that any problem-solving influenced by, or derived from, folklore will result in human-to-snake conflict. The stories recalled by community members were often emotionally charged, a theme noted in other contexts experiencing similar human-to-snake violence [10,29]. Recognising the commonality of this nexus between snake misconceptions, fear, and human-to-snake violence is crucial as it provides a clear mechanism to break down in future interventions aiming to minimise snakebites and encourage human-snake coexistence.

### How do Rubirizi community members consider their attitudes and behaviours toward snakes have changed since engaging with snakebite prevention workshops?

The majority of workshop attendees reported a change in their attitudes and behaviours towards snakes. This included granting snakes more respect, peace or space, and engaging in less human-to-snake violence. This may have been because workshops directly attempted to cultivate empathy with snakes. Participants were asked about the benefits of snakes and explored snake behaviour and biology to better understand snakes’ need for shelter, sunlight, food, and water. Delivery focused on co-creation activities allowing community members to fully engage in knowledge development, rather than passively receiving information. The workshops created a narrative regarding human-to-snake interactions, which was perhaps more memorable than a simple educational session. Across the literature, interventions aiming to change individuals’ attitudes and beliefs appear more effective in prompting behaviour change than simply sharing information. This holds true for topics such as antimicrobial resistance [44,45], zoonotic risk reduction [46], rabies [47], and malaria [48,49]. A pilot study has also been carried out in Eswatini, showing how a musical intervention generated community engagement to promote snakebite awareness and avoidance [50]. Unfortunately, a wider range of studies looking at snakebite prevention in the context of influencing local attitudes is lacking in the current literature. However, other research demonstrates that educational interventions have been successful in changing local practice when managing snakebite victims [51,52].

It appears that narrative snake bite prevention workshops can have impact. When interviewed, attendees self-reported a decreased likelihood of attempting to kill a snake upon encounter but rather ‘leave it at peace’. By narratively exploring dynamics of human and snake behaviour, workshops appear to have changed the attitudes of the Rubirizi population towards snakes, which directly impacts snakebite risk factors such as human-to-snake violence. Nevertheless, attitudinal changes described by workshop attendees were not replicated across workshop non-attendees who had only heard about the workshops from another community member, with most of these participants describing still attempting to kill snakes if they came across them. We believe this reflects the value of lengthy face-to-face and participatory workshops, which enable personal involvement in the developing narrative about snakes that in turn prompt shifts in attitude and reported practice. Indeed, behaviour-change research demonstrates that distributing knowledge points alone is unlikely to result in lasting change [53,54].

Intrinsic fear of snakes appears unshifted by the workshops. Attendees describe how they now engaged in peaceful behaviors toward snakes *despite* their fear, or *because* of their fear. Many suggest that because they feared snakes, they would adopt whichever behaviours they believed were optimal to avoid snakebites. It is important to reflect on this in terms of workshop aims: the delivery team were not aiming to reduce fear itself, as inherent fear of potentially dangerous species is beneficial [55]. Rather, workshops aimed to reduce human-to-snake violence that puts both humans and snakes at risk. This analysis suggests is that it is not simply a lack of information that drives fear of snakes, though it may be a contributor, but that local stories have a significant impact on fearful attitudes towards snakes. Asking participants to recount snake stories unearthed visceral, intrinsic beliefs that community members have surrounding snakes, and provided a basis for the origin and spread of these beliefs. By understanding and working with these beliefs, we can better promote human-snake coexistence, minimising human-to-snake violence and snakebites. Since stories are at the heart of information spread, and influence the community’s perceptions of snakes, we believe attitudinal and behavioral change can be instigated and encouraged with narrative-based interventions.

### Strengths and limitations of the study

This was a single site study, performed in the Rubirizi district of Southwest Uganda, hence not directly generalisable to wider populations. However, efforts were taken to ensure findings were robust. With 21 adult community members interviewed, the study features a relatively large sample size. The team included a trusted member of the community (YB) who speaks major local languages and dialects to conduct the interviews. This aimed to reduce participant bias by providing space for individuals to be more open in their interview responses. Secondary analysis of transcript data was conducted by researchers who were not present during the interviews (DMD, JM), minimising the risk of confirmation bias when interpreting findings. The main analyst (DMD) was not part of the study’s original development, hence came to the analysis process with no preconceptions regarding the data, further minimising the risk of confirmation bias in this secondary analysis. Two researchers developed the coding frame, conducted and cross-checked the analysis when deciding on themes, sub-themes, codes, and interpretations of these in relation to research questions. The wider team fed back on drafts to ensure both tone and meaning was captured from the interviews with YB, providing specific clarifications on any language which was mistranslated. However, it is important to note that this is a secondary analysis and the original study did not aim to explore the role of stories in such detail. Although the function of this study is not a pre- and post-intervention evaluation of the workshops themselves, it is important to note that no baseline data was collected prior to snakebite prevention workshops. This was due to COVID-19 lockdown and travel restrictions which delayed the project’s start and constrained delivery timeframes. When collecting interviews, we attempted to compensate for this by interviewing participants who had not attended workshops nor spoken to workshop attendees as their responses effectively functioned as a baseline for attitudes. Of course, key messages may have circulated through the community without conscious linkage to the workshops, and in this secondary analysis, we are aware that many stories and their interpretations may have been influenced by workshop discussions. However, the attitudes of non-attending interviewees appear very different to those who attended a workshop, and we feel this is important to discuss in terms of our third research aim.

### Directions for future research

Recognising the impact of community stories on attitudes to snakes is hugely informative. If storytelling has such a powerful influence on attitude and resulting behaviour, could this be harnessed to promote behavioural changes and minimise human-to-snake conflict and snakebite risk? This question forms the basis of the WHO storytelling handbook that supports practitioners to build effective stories to challenge attitudes and change behaviours around any topic important to a focal community [56]. A clear direction for future research involves creating and integrating stories into the community interventions which seek to promote human-snake coexistence. The workshops piloted by this team have already taken a participatory approach aimed at promoting tolerance between the Rubirizi community and snakes. However, while analysing this data we see the value of the narrative we created within the workshop structure. The team now wish to re-design workshops with explicit focus on creating narratives during the co-creation activities so that community knowledge and experiences can be re-framed in context of human-to-snake coexistence. We hope this focus on re-writing a community’s snakebite story ensures key points around minimising human-to-snake conflict remain memorable and spread effectively to non-attendees.

## Conclusion

This secondary qualitative analysis of interview data explores the role of stories in community attitudes toward snakes in rural South-Western Uganda. We show that most community stories place snakes in positions of power with dangerous, fear-inducing, manipulative intent. They often portray snakes in supernatural roles where attributes differ from natural snake behaviours. These stories appear to influence community attitudes toward snakes. Despite reporting relatively low incidents of snakebite fatalities, communities hold fearful attitudes toward snakes reminiscent of their roles in stories and use this to justify human-to-snake violence. However, participants who have attended a snakebite prevention workshop appear to soften their attitude. Although workshop attendees still report fearing snakes, they discuss having respect for these animals and are less likely to suggest harming them. We consider this analysis a demonstration of the power of community narratives. We suggest future snakebite prevention research projects should take time to comprehensively understand existing local narratives before designing interventions. This will allow the basis of current snake-related attitudes to be understood and facilitate the development of locally appropriate interventions which explore misconceptions. We also advocate for a narrative approach to interventions, using participatory and co-production methods to allow communities to re-write their own stories of snakebite prevention and human-snake coexistence.

## Supplementary Material

Supplementary file.docx
