# Supplementary material for: A double narrative: stories underpin fearful community attitudes toward snakes but offer a mechanism to reduce human-to-snake conflict
Source: Glob Health Action. 2026 May 5;19(1):2655948. doi: 10.1080/16549716.2026.2655948 (PMC13148094; doi:10.1080/16549716.2026.2655948)
Supplement: Supplementary file.docx [file ZGHA_A_2655948_SM1058.docx]

# Supplementary file

## 1. Snake bite interview questions (English)

- Question 1: Did you attend one of the snakebite prevention workshops?
- Question 2: How often do you see snakes around your community?
- Question 3: Do you know what kind of snakes you have in your community?
- Question 4: What do you think about snakes?
- Question 5: Do you think snakes have any positive or useful qualities?
- Question 6: Do you know any folktales, stories, or other tales involving snakes?
- Question 7: Is there anything you have been told about snakes that you don’t believe?
- Question 8: Do (any) snakes have a special cultural significance to you or your community?
- Question 9: Do you know anyone in your community who has been bitten by a snake?
- Question 10: What would you do if you came across a snake?
- Question 11: What you do or someone else was bitten by a snake (especially if you believed the snake to be dangerous)?
- Question 12a: Is there anything you normally do as a way of avoiding snakes or preventing being bitten by them?
- Question 12b: Do you know of anything others do to prevent snakebite?
- Question 13 (If participant attend workshop only): Can you remember any of the things we discussed to prevent snakebites in the workshop?
- Question 14: Is there anything else you think we might be interested to know about snakes and snakebites in your area?

## 2. Snake bite workshop: Facilitation Guide

### Session aims:

- Understanding basic snake biology and behavior
- How to minimize human-snake interactions
- Knowledge exchange – community have information to give the research team and we are here to listen to this

### Time scale:

4 -5 hours including a break

### Materials needed:

- Large private room in community space (school, health center etc.)
- Printed consent forms and pens
- Posters
- Chalk and board or other writing equipment that is easy to see
- Drinking water
- Handwashing stations
- Food if having a lunch break
- Laptop or projector and screen to show video

### Personnel needed:

- 1 facilitator
- 1 co-facilitator
- 1 observer

Facilitation team requirements: local language skills, background or training on snake biology and behavior, community engagement skillset

### Outline of programme:

- Introduction to snake behavior and biology
- What do we know about snakes and how can they be useful to us?
- Locating Snakes
- Short break or lunch break depending on time
- Community generated solutions – group work
- Discussion
- WHO video
- Q&A
- Close

### Important tasks to do before the start of the workshop

1. Sign consent forms
2. Discuss aims of workshop
3. Option to withdraw/leave
4. Fire drill/ health and safety messaging

### Introduction to snake behavior and biology

Aims:

- Discuss with the community how snakes are different to other animals and use this information to understand snake behaviors.
- Realize that people and snakes often want the same things, and this can lead to conflict where people get bitten and snakes get killed
- Understand that snakes are delicate animals and want to avoid people if they can.

### Presentation by facilitator:

1. Snakes are different from birds and mammals
2. One key difference is that snakes cannot regulate their body temperature internally, so they have to take heat/cool from the sun or environment around them.
3. If they’re body temperature is right, they will move around and likely be seen by people, they will also move quickly away from you
4. If it is too cool the snakes need to hide because they cannot move fast enough to escape predators or find prey to eat
5. If they are too hot, they can die (like people) so in hot countries like Uganda snakes need to find shade when it is hot
6. Snakes and people generally want the same thing
7. Shade when it is hot
8. Sun when it is cold
9. Conflict can occur between snakes and people because we want the same things

Open question: What do you do when it is hot?

Purpose of Q is to build connection between what people and snakes both do

Example answers

- Bathe
- Shade
- Go in the house
- Trees/bushes

Key message: Snakes also seek out these shaded options when it is hot – this can bring people and snakes into contact and cause problems (risk of bites, risk of people killing snakes). We want to keep people and animals safe

1. Snakes are very delicate
2. Basic skeletal info
3. Need to find safe places to hide
4. Want to avoid conflict with humans as it is harmful to them
5. However, if snakes feel threatened, they will try to defend themselves and this includes biting people

Question: how would you feel if you were threatened?

- Feel scared
- Try to protect yourself
- Try to run away
- Snakes will do all of this too
- Dangerous snakes can kill with a bite

Key message: This is why snakes can bite people. Snake bites can be very dangerous but they only happen because the snakes are trying to protect themselves.

1. Snakes also need to drink and seek out water
2. This is another reason people and snakes come into contact – because they are both looking for water to drink and stay cool
3. Spaces around water and good hiding places for snakes

### Key messages

- The reason snakes and people come into contact is because they are often looking for the same things (shade, heat, water)
- Snakes are also very delicate, so they need to defend themselves if threatened

### What do we know about snakes?

Aims:

- To discuss how snakes can benefit or be useful to people.
- To understand the range of snakes we have in Uganda
- To understand that not all snakes are dangerous

Question: How can snakes be useful/beneficial to people/us?

List actions from community on the board

Key benefits which may need adding from the facilitator team

- Venom used to make medicines
- Most snakes eat rats/mice – so very good for keeping vermin out of your house and away from livestock
- Minimize infection
- Minimize food loss/home damage

Key messages

- Snakes are beneficial to us so we shouldn’t try to kill them
- Trying to kill snakes is very dangerous as it often means we get close enough to be bitten
- Demonstrate this – helps participants to understand the need to keep distance
- If a snake has a way to escape – just let it go
- The snake will want to move away from you, it will not want to stay close to you
- We are training people in this community to safely move snakes away from homes
- If the snake remains close to you, or you are worried about it coming back you can call these people and they will safely remove the snake

Types of local snakes

Question: How many snakes do you have in this area:

Ask people to shout out names and tally on the board.

- Can then count the numbers and ask people if there are more – how many more? Try to get a final number.

Answer is 48 (use poster to show this)

- Ask how many are dangerous

Answer is 11 are dangerous (use poster to show this)

- Are people surprised by this?

Key messages

- Most snakes around our homes are harmless and provide all the benefits we discussed earlier
- However, the dangerous snakes are very dangerous so if you are bitten you should always assume it is an emergency and seek help from your healthcare professional.

### Locating snakes

Aims:

We will now put our learning into practice by taking a walk to see if we can spot places that we think snakes might like to hide, and if we can do anything to make these situations safer for us and the snakes.

Activity:

- Take group outside and walk around the venue
- Ask group to identify places where they usually see snakes
- Ask where snakes might want to hide based on the biology/behavior points we have discussed
- When a location is identified discuss ways to minimize human-snake contact here

Examples of locations where snakes might be found

- Under rocks/leaves
- In holes in the housing structure
- In animal houses
- In pipes
- Close to water sources
- Basking in the sun if it has been cold
- Under log piles
- Near food/water
- In beds

Examples of mitigation options

- Move piles of rocks/wood future from the house (at least 2m)
- Check before putting hands under leaves/rocks/logs etc.
- Wear sturdy footwear when walking through long grass, leaves or where you have limited visibility
- Good home maintenance – filling in holes, mesh over pipe entrances
- Good lighting outside (solar or torches)
- Door lips to prevent easy access of snakes
- Regularly cleaning home and animal houses
- Use bed nets and keep them tucked in
- Riase beds off the floor if possible
- Keep beds away from doors and temporary walls

### What do we do to better manage human-snake interactions?

Aims:

To understand what feasible solutions to human-snake interactions are possible in this community and to allow the community to share their knowledge.

Activity

- Write the following 3 headings on the board
- Ask people to work in groups of 5-6 to answer the questions
- Spokesperson shares answers to group and the board is annotated

| **What are we doing now** | **What could we be doing** | **Why aren’t you doing it?** |
| --- | --- | --- |

Reflection questions

- What is stopping you from doing some of the ‘could’ behaviors – is this access to equipment etc?

Key messages to avoid human-snake interactions and snake bites

- Bed nets are very helpful to avoid snake bites and many other health issues - suggest this is a very key item to invest in
- Move piles of rocks/wood future from the house (at least 2m)
- Check before putting hands under leaves/rocks/logs etc.
- Wear sturdy footwear when walking through long grass, leaves or where you have limited visibility
- Good home maintenance – filling in holes, mesh over pipe entrances
- Good lighting outside (solar or torches)
- Door lips to prevent easy access of snakes
- Regularly cleaning home and animal houses
- Use bed nets and keep them tucked in
- Riase beds off the floor if possible
- Keep beds away from doors and temporary walls

### WHO video

- Show 6 min WHO video
- Pause between each point for translation
- Ask if any questions
- Answer as best as possible
- Aim to avoid direct do’s and dont’s on first aid
- Be open to local knowledge, myths and traditions about snakes – do not say it is wrong

### Finisher – ‘Even better if’

- Write on the board ‘even better if’
- Ask the participants what could be better about the workshop
- Write all points on board and tell participants this will be recorded and listened to
- Thank you and goodbye – stress a key point that the facilitation team have learned.

## 3. Table of participant demographics

| **Transcript Number** | **Gender** | **Age** | **Education level completed** | **Meaning of education** | **Workshop attended?** | **Heard of workshop from word of mouth?** |
| --- | --- | --- | --- | --- | --- | --- |
| 1 | F | 25-30 | p7 | Completed Primary School | yes | NA |
| 2 | F | 28-34 | S4 | O levels | no | yes |
| 3 | F | 30-38 | P6 | Did not finish primary school | no | no |
| 4 | F | 30-34 | S4 | O levels | yes | NA |
| 5 | M | 30-34 | Diploma | Second year of university | no | no |
| 6 | M | 28-35 | S3 | pre O levels | yes | NA |
| 7 | F | 25-38 | S3 | pre O levels | no | no |
| 8 | F | 20-30 | P3 | Did not finish primary school | yes | NA |
| 9 | F | 35-40 | S4 | O levels | yes | NA |
| 10 | F | 30-45 |  | pre O levels | yes | NA |
| 11 | F | 30-35 | P7 | Completed Primary School | yes | NA |
| 12 | F | 25-35 | P7 | Completed Primary School | yes | NA |
| 13 | M | 18-25 | A level |  | No | Yes |
| 14 | F | 25-30 | S3 | pre O levels | Yes | NA |
| 15 | M | 25-28 | Degree | Grade 3 primary teacher | No | Yes |
| 16 | M | 39 | P6 | Did not finish primary school | No | Yes |
| 17 | F | 37-40 | P6 | Did not finish primary school | No | Yes |
| 18 | F | 27-30 | P7 | Completed Primary School | No | Yes |
| 19 | F | 40-50 | P7 | Completed Primary School | Yes | NA |
| 20 | F | 20-30 | S4 | O levels | No | Yes |
| 21 | M | 45-50 | P5 | Did not finish primary school | No | Yes |
